# Appraisal of the Role of Gaseous Signaling Molecules in Thermo-Tolerance Mechanisms in Plants

**DOI:** 10.3390/plants13060791

**Published:** 2024-03-11

**Authors:** Harsha Gautam, Sheen Khan, Nidhi, Adriano Sofo, Nafees A. Khan

**Affiliations:** 1 Plant Physiology and Biochemistry Laboratory, Department of Botany, Aligarh Muslim University, Aligarh 202002, India; 2 Department of European and Mediterranean Cultures: Architecture, Environment, Cultural Heritage (DiCEM), University of Basilicata, 75100 Matera, Italy

**Keywords:** gaseous molecules, stress resistance, heat stress tolerance

## Abstract

A significant threat to the ongoing rise in temperature caused by global warming. Plants have many stress-resistance mechanisms, which is responsible for maintaining plant homeostasis. Abiotic stresses largely increase gaseous molecules’ synthesis in plants. The study of gaseous signaling molecules has gained attention in recent years. The role of gaseous molecules, such as nitric oxide (NO), hydrogen sulfide (H_2_S), carbon dioxide (CO_2_), carbon monoxide (CO), methane (CH_4_), and ethylene, in plants under temperature high-temperature stress are discussed in the current review. Recent studies revealed the critical function that gaseous molecules play in controlling plant growth and development and their ability to respond to various abiotic stresses. Here, we provide a thorough overview of current advancements that prevent heat stress-related plant damage via gaseous molecules. We also explored and discussed the interaction of gaseous molecules. In addition, we provided an overview of the role played by gaseous molecules in high-temperature stress responses, along with a discussion of the knowledge gaps and how this may affect the development of high-temperature-resistant plant species.

## 1. Introduction

Planet’s average temperature change is increasing the global average temperature, resulting in higher temperatures and more intense rain. Moreover, temperature is one of the most important environmental factors influencing crop development and distribution [1]. specific. Plants respond to changes in their environment by changing the expression of specific genes, which can affect physiological and metabolic processes [2]. Between 1951 and 2012, the Earth’s average surface temperature increased by approximately 0.72 °C due to the rise in greenhouse gas emissions caused by human industrial development. If this situation remains unchanged, it is predicted that the average global surface temperature will increase by 3.7 ± 1.1 °C by the end of the twenty-first century [3]. Therefore, adverse, environmental conditions significantly hinder the growth and development of plants, preventing them from reaching their full genetic potential and reducing yield. The majority of crop losses worldwide and a more than 50% decline in the average yield of most crops are attributed to abiotic stresses [4]. Heat stress has emerged as one of the most serious and widespread abiotic stresses that can impede agricultural production due to its impact on crop plants growth, development, and yield. The frequency of heat stress, on the other hand, varies significantly among climatic zones and is dependent on the duration and probability of high temperatures as well as the timing of diurnal plants during high temperatures. Global warming, caused by the rapid and increased emission of greenhouse gases like NO_2_ and CO_2_ from various industries and automobile sources, is responsible for the daily rise in average global temperature. Additionally, there are variations in how different plant species and developmental stages respond to high temperatures [5,6,7]. Researchers are gaining interest in identifying compounds that have the potential to protect plants against the negative effects of heat stress. [8,9,10]. Research publications on heat stress in plants showed a notable increase from 2013 to 2023.

Gaseous molecules are currently regarded as significant signaling mediators [11,12,13,14]. Plants under high temperatures have benefited from exogenous application of gaseous molecules, osmoprotectants and phytohormones primarily because of their growth-promoting and antioxidant properties [9,15,16]. According to the most recent research, gaseous molecules have a significant amplifying ability to enhance plant adaptive responses that is still largely unexplored in crop production [13,14,17]. Small gaseous molecules produced by living organisms and serving as signaling agents are referred to as “gasotransmitters”. These molecules can cross cellular membranes; they don’t require a particular receptor to function and can be produced by enzymes. Additionally, they interact closely with other signaling mediators and react with specific cellular target components [17]. The main gasotransmitters in plants are nitric oxide (NO), carbon dioxide (CO_2_), hydrogen sulfide (H_2_S), and carbon monoxide (CO) [14]. Currently, gasotransmitters are also being considered for methane (CH_4_) and the gaseous phytohormone ethylene (C_2_H_4_). The characteristics of ethylene action are not entirely consistent with the concepts of gasotransmitters, and methane’s physiological uses and the mechanisms underlying their synthesis in plants have not yet been thoroughly investigated [14,18,19]. Methane’s inclusion is still unclear despite the fact that few reports highlight its potential value as a signaling molecule [17]. However, Yao et al. [14] recently reported that in addition to NO, CO, and H_2_S, CH_4_ is a gasotransmitter involved in the response of plants to abiotic stress. Additionally, gaseous molecules are particularly crucial in helping plants adapt to unfavorable environmental conditions [13,14].

The plants’ ability to generate gaseous molecules in response to abiotic stresses has been shown [13,14,20]. In contrast to other abiotic stresses, all of the gaseous molecules mentioned above control the response to high-temperature stress [21,22,23,24,25]. Exogenous application of gaseous molecule donors mediates a range of plant responses to high-temperature stress, including photosynthesis, oxidative defense, osmolyte accumulation, gene expression, and protein modifications [17,23,26,27,28,29]. High-temperature stress alters the expression of genes involved in direct heat stress protection, such as those for osmoprotectants, detoxifying enzymes, transporters, and regulatory proteins [30,31]. There have been several review articles on the biology of abiotic stress connected to high-temperature stress in plants, but in-depth studies of the relationship between gaseous molecules and high-temperature stress in plants are lacking. Here, we give a thorough overview of the mechanisms governing the regulation of thermo-tolerance by gaseous molecules and highlight their unique features.

## 2. Gaseous Molecules

Gaseous molecules are small molecules which are produced by living organisms and are used for carrying biological signals. Research on gaseous molecules is advancing quickly, and we are learning more about their potential applications in the fields of science and medicine [32]. Particular biological processes are regulated by gaseous molecules, which include ethylene, NO, H_2_S, CO_2_, CO, and CH_4_. The extensive study and analysis of endogenous gaseous molecules emissions in plants has recently aided our understanding of new signaling pathways. According to earlier studies, plants typically produce these molecules in response to abiotic stress [20,29,33]. Furthermore, growing evidence shows that gaseous molecules can play an essential role in increasing plant tolerance [34,35,36]. Table 1 summarizes a few studies that examine how plants respond when exposed to high temperatures and exogenous gaseous molecules.

### 2.1. Ethylene

Ethylene is the most basic unsaturated hydrocarbon. It affects of various plant growth and developmental processes, including germination, leaf and flower senescence and abscission, cell elongation, fruit ripening, nodulation, and stress response [53]. Internal signals control ethylene production during development and in response to external stimuli like biotic and abiotic stresses [54,55,56]. Two specific enzymatic processes contribute to the synthesis of the ethylene pathway, which is relatively simple. The enzyme ACC synthase (ACS) transforms the substrate S-adenosyl-l-methionine (SAM) into ACC and 5′-methylthioadenosine (MTA) in the first step [57,58]. The enzyme ACC oxidase (ACO) converts ACC into ethylene, CO_2_, and cyanide in the second step [59,60]. By converting to β-cyanoalanine, a set of β-cyanoalanine synthases quickly reduces the toxicity of the cyanide by-product [61,62].

Ethylene stress, despite being essential for plant survival and adaptation in the face of environmental challenges, ethylene ultimately causes plant mortality. Depending on whether ACS activity has been induced or suppressed, heat stress can either stimulate or reduce ethylene production. Heat stress generates a substantial buildup of ROS, resulting in oxidative stress. When ROS levels reach a certain threshold, it signals the start of ethylene production. Together with ethylene, oxidative stress, particularly caused by hydrogen peroxide (H_2_O_2_), causes leaf senescence and chlorosis under heat stress. At the same time, ethylene-induced H_2_O_2_ buildup increases ethylene synthesis. Through numerous levels of regulation, ethylene of impacts a plant’s ability to cope with various environmental stresses [63]. In creeping bentgrass, ethylene preserves cells’ structural integrity and stability and plays a crucial role in thermotolerance [64]. Heat shock proteins enhance *ERF1* overexpression in Arabidopsis and improve heat tolerance in transgenic lines over wild type by up-regulating the expression of heat tolerance genes [65]. The conferring of thermotolerance by ethylene-mediated signaling also assists in of heat shock factors in rice seedlings [37]. Furthermore, by lowering oxidative stress or engaging genes associated with ethylene signaling in plants, ethylene signaling promotes heat resistance and preserves chlorophyll content [37]. The ethylene response factor (*ERF021*) has a significant 78.7-fold initiation during heat stress [66], indicating ethylene’s role in soybean tolerance to heat stress. Heat stress activates numerous ethylene-responsive genes (*ER5, ER21, LeJERF1, and ER24*) in developing pollen grains of *Solanum lycopersicum* [67]. The regulation of stress-specific genes by *ERF1* could enhance way plants tolerate heat, drought, and salt stress [65]. The increased CO_2_-induced heat stress response in tomato plants could be attributed to the up-regulation of genes involved in ethylene biosynthesis and signaling and the subsequent induction of HSPs [68]. ACC (ethylene precursor) pretreatment increased the survival rate of Arabidopsis at 40 °C, reduced oxidative damage, and induced basal thermotolerance [69]. Additionally, previous work has shown that the Arabidopsis *ein2* and *etr1* ethylene-signaling mutants are defective in basal thermotolerance [70]. After exposure to ethephon, defensive mechanisms against oxidative stress were enhanced (including increased levels of glutathione-disulfide reductase, glutaredoxin, and protein disulfide isomerase) in tomato [24]. When rice seedlings were exposed to heat stress, ethylene-mediated signaling helped to reduce oxidative damage, maintain chlorophyll levels, and improve thermotolerance [37]. Contrarily, AVG, an ethylene inhibitor, was applied exogenously to creeping bent-grass leaves, which may have prevented leaf senescence by up-regulating antioxidant enzyme activity and reducing ethylene production [71].

Under both favorable and adverse conditions, studies have revealed that ethylene may play a role in synthesizing secondary metabolites, osmolytes, and antioxidant metabolism to modulate environmental stress tolerance [72]. According to in vitro studies, *Glycine betaine* shields the D1/D2/Cytb559 complex of photosystem II from heat stress (35 °C) [73]. Furthermore, proline accumulation under heat stress partially blocks ethylene production; as a result, low levels of ethylene may be a factor that increases sensitivity to heat caused by proline [74]. According to a recent study by Ma W. et al. [75], melatonin predominantly affects secondary metabolite biosynthesis and plant hormone signal transduction pathways through ethylene. This indicated that ethylene has a role in plants’ ability to produce secondary metabolites. According to Watkins et al. [76], ethylene controlled the accumulation of flavonol in guard cells, indicating that flavonol, in its capacity as an antioxidant, adversely regulates stomatal closure by scavenging ROS under stressful circumstances. Heat stress, on the other hand, can disrupt carbohydrate metabolism, which can impede plant growth and development, sterilize pollen, prevent fertilization, and reduce grain output in various crops. The impact of ethylene on sugar metabolism is well understood, particularly in the context of senescence or fruit ripening, although it has received less attention when heat stress is involved [77,78]. Soluble sugars function as signal molecules to control several photosynthesis-related gene expressions and either act as direct antagonistic signals or alter cellular signaling pathways to trigger stress response signals and boost plant stress resistance [79,80].

**Figure 1:**
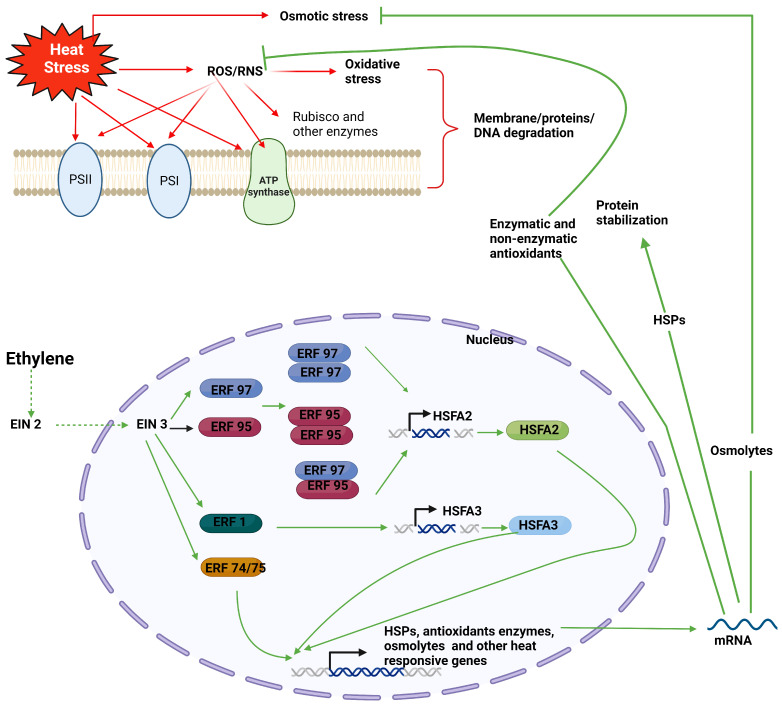
Heat stress can directly disrupt or harm the functioning of proteins, membranes, and DNA in plants or by generating reactive oxygen and nitrogen species (ROS/RNS). This disruption negatively impacts photosynthesis and plant growth. Ethylene, acting through EIN3 (signaling component), triggers the activation of ERF95, ERF97, ERF1, ERF74, and ERF75 by binding to their respective promoter regions. ERF95 and ERF97 can form both heterodimers and homodimers, and they play a role in regulating the expression of HSFA2 by directly binding to its promoter. ERF1, on the other hand, controls the expression of HSFA3. All of these, in turn, regulate the expression of various genes responsible for producing heat shock proteins (HSPs), antioxidants, and osmolytes, thus helping plants in heat stress tolerance. Red solid arrow: Direct heat-mediated effect; Red faded arrow: Heat-induced ROS/RNS mediated effect; Green arrow: Ethylene mediated responses (solid arrow: direct pathway; dotted arrow: multistep pathway; head flat arrow: Inhibition).

According to Wang et al. [81], ethylene considerably raises the fructose and glucose levels in ripe jackfruit but does not affect sucrose and total soluble sugar contents [82]. In cotton plants exposed to heat stress, 1-MCP treatment reduced the amount of soluble sugar content [83]. After AVG application to bentgrass plants, monosaccharides like glucose or fructose levels dropped in response to heat stress [64]. According to Paraankusam et al. [10] and Ali et al. [84], the buildup of ROS and RNS is a significant factor in regulating plant responses to heat stress. When plants are exposed to heat stress, ROS and RNS production often increase quickly, although this depends on the extent and duration of the heat exposure [85,86]. Ethylene could modulate the activity of antioxidant enzymes, which regulates ROS metabolism [87]. When creeping bentgrass (*Agrostis stolonifera*) shoots were exposed to heat stress (35 °C), foliar treatment of ACC (100 µM) did not alter the tendency for CAT and APX activity to decline; however, it did increase the activity of SOD and POD [88]. Under heat-stress conditions, ethylene plays a significant role in ROS metabolism. Figure 1 depicts a schematic representation of the impacts of heat stress on photosynthesis and plant growth, as well as ethylene-mediated heat stress tolerance in plants.

### 2.2. Nitric Oxide

Nitric oxide (NO) is currently recognized as a crucial signaling molecule in plants, where it controls a wide range of cellular processes involved in growth, development, and environmental interactions. NO is a small, diatomic gas that has no charge is colorless and can diffuse quickly across membranes [89,90]. NO is involved in seed germination, root development, stomatal closure, pathogen challenge, plant reproduction, and stress responses [91,92,93,94,95]. In plants, NO synthesis occurs through two fundamental processes: the oxidative or arginine-dependent system, and NO synthesis occurs through two key processes: the oxidative or arginine-dependent system and the reductive or nitrite-dependent pathway. The two oxidative mechanisms that produce NO and have received the most focus are the oxidation of L-arginine and polyamines. The enzymatic activity of nitric oxide synthases (NOSs) mediates the L-arginine-dependent pathway. L-arginine first combines with molecular oxygen to form N-hydroxy-L-arginine, which is then transformed into citrulline with the production of gaseous NO. The nitrogen atom of the resulting NO molecule derives from the guanidine group of arginine, while the oxygen atom originates from an oxygen molecule involved in the reaction [96,97]. The nitrate/nitrite reduction processes that occur with the involvement of enzymes capable of catalyzing the one-electron reduction of nitrite to NO are related to the reductive routes of NO production. In the apoplast of the barley aleurone layer, non-enzymatic nitrite-dependent NO production was found to occur at low pH and high nitrate concentrations [98,99,100]. The mitochondrial electron transport chain (mETC) participates in the enzymatic reductive NO generation in the cytoplasm, chloroplasts, peroxisomes, and mitochondria of plant cells [101,102].

Plants can be protected from heat stress by NO acting either directly as an antioxidant and scavenging ROS or as a signal molecule to induce thermotolerance by up-regulating the expression of heat-responsive genes. In plants, NO has emerged as a critical signaling molecule that activates ROS-scavenging enzymes in response to abiotic stresses such as heavy metal, drought, heat, and salinity stress [103,104,105]. In previous studies, heat stress has been found to increase NO content in wheat, and exogenous NO application improves thermo-tolerance in *Lablab purpureus* and wheat [106,107,108]. Similarly, rapid NO generation was observed during heat stress in tobacco [109]. High temperatures (38 °C) reduce S-nitrosoglutathione reductase (GSNOR) activity in plants, leading to decreased nitric oxide (NO) levels and increased oxidative damage due to intensified tyrosine nitration [10]. The significance of the *GSNOR* gene has been well-documented through null mutations (*gsnor1-3 and hot5-2*), which result in truncated GSNOR protein expression in Arabidopsis, leading to NO hyper-accumulation and heightened heat sensitivity. This underscores the crucial role of maintaining an optimal NO level for plants to develop thermotolerance. In response to thermal stimuli, the activation of the S-nitrosoglutathione reductase (GSNOR1) encoding allele, hot temperature 5 (HOT5), leads to elevated levels of S-nitrosothiols and nitrate, which undergo conversion to NO due to the transcriptional induction of chloroplast phosphoenolpyruvate/phosphate translocator/arginine amidohydrolase-1 genes which causes S-nitrosylation of cysteine residues in *NPR1* genes. This process results in the S-nitrosylation of cysteine residues within *NPR1* genes, which is crucial for detecting and mediating heat stress responses in plants [10]. The activated *NPR* genes interact with ROS-dependent systemic signaling, leading to the induction of RBOHD protein, which generates free radicals [107]. This process triggers NO-mediated S-nitrosylation of proteins involved in ribosome assembly and translation in the chloroplast, pathways related to enhancing cytokinins and ABA initiating downstream defense signaling for heat stress adaptation, and various transcription factors like WRKY and MYB, ultimately inducing the expression of molecular chaperones to mitigate protein denaturation and maintain cellular homeostasis during heat stress [107].

Studies on NO-mediated thermo-tolerance show significant variation, and the relationship between NO production and stress resistance is not well-established. Abscisic acid (ABA) has been found to cause H_2_O_2_-dependent NO formation, whereas ABA and H_2_O_2_ mediated increase in MAPK and antioxidant gene expression depends on endogenous NO generation [110,111]. NO-mediated thermo-tolerance requires ABA.

**Figure 2:**
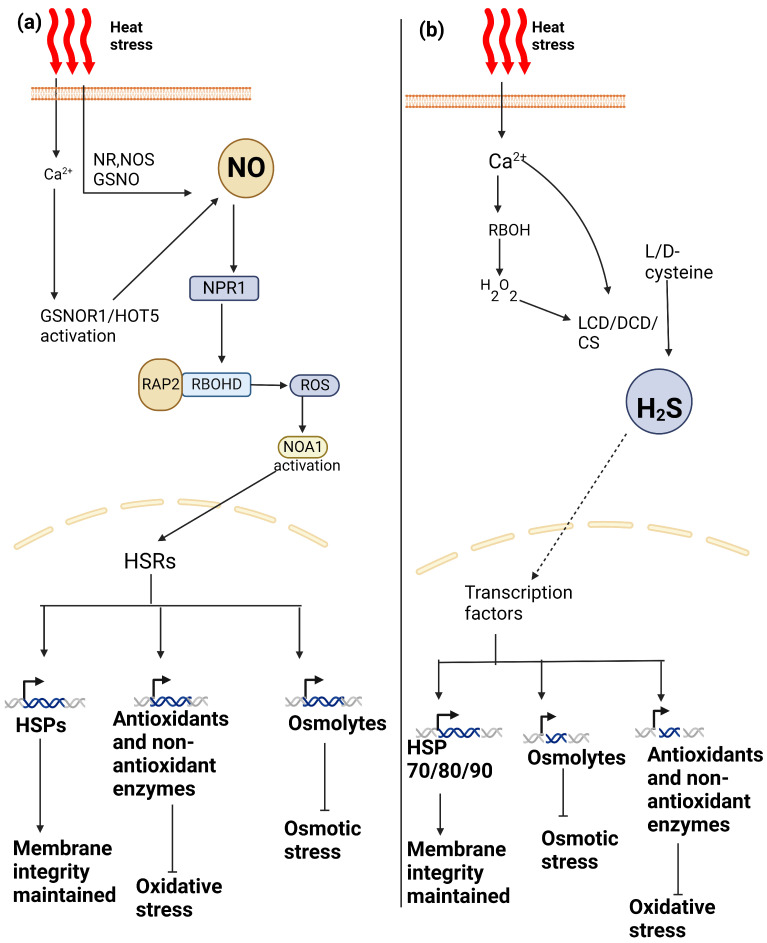
(**a**) Heat stress triggers nitric oxide (NO) production and increases calcium levels. Increased calcium concentration through its sensor proteins activates genes *GSNOR* (S-nitrosoglutathione reductase) and *HOT5* (hot temperature 5), thus fine-tuning NO levels. NO leads to S-nitrosylation of cysteine in *NPR1* (non-expresser of pathogenesis-related), mediating heat stress responses. ROS-driven systemic signaling converges with *NPR* genes, activating RBOHD (respiratory burst oxidase homolog D) via related to apetala 2 (RAP2). This triggers *NOA1* (NO-associated) genes, initiating heat stress responses (HSR), including oxidative defenses, osmolyte accumulation, and heat shock proteins (HSPs). (**b**) The heat stress signal is detected at the plasma membrane by a sensor. This signal is then relayed through calcium (Ca^2+^) and hydrogen peroxide (H_2_O_2_) signaling pathways, resulting in the activation of L-cysteine desulfhydrase (LCD), D-cysteine desulfhydrase (DCD), cysteine synthase (CS) for hydrogen sulfide (H_2_S) production. H_2_S interacts with transcription factors, driving the transcription of antioxidants, osmolytes, and heat shock proteins (HSPs), thereby enhancing cell thermotolerance.

Inducing antioxidant enzymes, as well as lowering ion leakage, ROS levels and peroxidation of membrane lipids, is how exogenous NO causes heat tolerance in a variety of plant species. In *Oryza sativa*, tobacco, Arabidopsis, *Festuca arundinacea*, and wheat, NO was found to have a protective effect against damaging effects caused by high temperatures [106,109,112,113,114]. It has been demonstrated that NO stimulates the expression of HSP-encoding genes. Under heat stress, NO protects chloroplasts from oxidative damage by increasing gene expression, producing small HSP26 [112]. NO, along with ROS, has been shown to play a role in the regulation of HSP70 synthesis and accumulation under heat stress [115]. Xuan et al. [116] discovered evidence for the role of NO in thermotolerance, and they found that NO, via boosting the DNA-binding activity of heat shock transcription factors (HSFs) and the accumulation of HSP18.2, positively contributed to thermotolerance in Arabidopsis. The role of NO in stress tolerance has been investigated using pharmacological experiments in which NO levels were altered using donors and scavengers, by employing mutants, or developing transgenic plants [117,118]. Exogenous or endogenous NO has been demonstrated in prior research to significantly enhance plant thermotolerance. NO was shown to significantly increase the thermotolerance of *Vicia faba* plants. The application of a NO scavenger, 2-4-carboxyphenyl-4,4,5,5-tetramethylimidazoline-1-oxyl-3-oxide (cPTIO), could significantly reduce NO production, demonstrating that NO is endogenously produced in plants during heat stress [119]. The application of NO donors, dramatically reduced the formation of oxidative stress indicators, such as increased H_2_O_2_ levels in wheat under heat stress [41]. Heat-induced damage was minimized in rice seedlings, and the survival rate of wheat leaves and maize seedlings was raised by NO pre-treatment [112,120]. Recent studies have also revealed an interaction between the activation of ROS-scavenging enzymes and, the regulation of heat-responsive genes and the alleviation of heat stress by exogenous NO [107,119]. According to Hossain et al. [121], NO may play a role in the decline of NPQ when exposed to heat stress. Increasing NO levels in plants has been shown to increase heat stress tolerance, which makes it a potential target for developing strategies to reduce the adverse effects of heat stress on plant growth and productivity. Overall, NO plays rolesa critical role in regulating plant responses to heat stress. (Figure 2a) depicts the numerous functions that NO plays in modulating plant heat stress responses.

### 2.3. Hydrogen Sulfide

With its distinctive smell of rotten eggs, hydrogen sulfide (H_2_S), a small colorless gas, has long been regarded as an environmental hazard [122]. H_2_S is uncharged, small, and highly diffusible, making it plausible that it could pass through the plasma membrane and those of organelles without the assistance of proteins like aquaporins [123]. Regardless of its intrinsic toxicity, hydrogen sulfide (H_2_S) is increasingly acknowledged as a crucial component of the group of small diffusible substances used by organisms in cell signaling [124,125,126]. As a key player in the regulating multiple processes regulating plant growth, development, and responses to environmental factors, H_2_S has emerged as a key signaling molecule. It’s significant to note that the biological effects of H_2_S in plants involve interactions and cross-talk with signaling pathways of other plant gasotransmitters as well as reactive oxygen and nitrogen species [127,128]. The multiple functions of H_2_S in interactions with phytohormones also influence the biological roles of H_2_S in plant growth, development, and responses to abiotic stressors. Phytohormones control endogenous H_2_S levels, but H_2_S also affects the synthesis, distribution, and signaling of a variety of plant hormones during physiological reactions [129]. Chloroplast, cytosol, and mitochondria are three subcellular spaces in plant cells where H_2_S is present and can be produced by enzymes involved in the metabolism of cysteine and sulfur. L/D-cysteine desulfhydrase, sulfite reductase, cyanoalanine synthase, cysteine synthase, and O-acetylserine(thol)lyase isoforms are some of the enzymes involved in H_2_S metabolism [130]. Chloroplast is a crucial component of endogenous H_2_S production in plants. Sulfite reductase found in chloroplasts, catalyzes the conversion of sulfite to sulfide during the sulfate reduction pathway [131]. H_2_S is metabolically produced in the cytosol from cysteine. Cysteine biosynthesis produces H_2_S as a byproduct, which is catalyzed by O-acetylserine(thiol)lyase (OASTL) enzymes [132,133]. There are two steps in the biosynthesis of cysteine: Serine acetyltransferase (SAT) first converts acetyl-CoA and serine into an intermediary product called O-acetyl-Ser (OAS), and OASTL then catalyzes the incorporation of sulfide into OAS to generate cysteine [132]. L-cysteine desulfhydrase (L-CDES) enzyme (specific for L-cysteine) and D-cysteine desulfhydrase (D-CDES) enzyme (specific for D-cysteine) release H_2_S from cysteine, which results in the production of pyruvate and ammonia [131,133].

Several studies have suggested that H_2_S plays a role in the tolerance to both high and low temperatures. H_2_S can influence plant’s ability to to respond to a variety of environmental stimuli under stress conditions by reducing stress-related damage and activating defensive systems [130,134,135] H_2_S accumulation under heat stress has been reported, and this’ may be connected to plants’ development of stress tolerance [25,136]. On the basis of hydrogen sulfide’s priming effect on plant redox signaling, antioxidant ability, and certain components of cellular defense, plants’ tolerance to abiotic stresses such as salinity, drought, heavy metal, and high-temperature stress can be increased [137]. H_2_S applied exogenously causes plants to cross-adapt to several abiotic stressors [138]. The ability of L-cysteine desulfhydrase (DES1) to function in response to abiotic stress carried on by the synthesis of H_2_S has been associated with increased tolerance to osmotic stress, heat stress, and drought [23,139,140]. H_2_S-mediated activation of carbonic anhydrase and OAS-TL activity provided evidence for the role of H_2_S in plant tolerance to dehydration stress, whereas both dehydration stress and an exogenous application of NaHS stimulated DES1 activity, which increased plant H_2_S levels derived from accumulated Cys [141]. It is known that H_2_S regulates the expression of genes essential for synthesizing phytohormones, which could change the relative amounts of hormone levels regulating various processes during plant growth and stress responses [129]. Proline levels are altered by H_2_S, and proline is crucial for maintaining redox balance and preventing oxidative stress [142,143]. H_2_S can raise glutathione levels, which is one of its beneficial effects [144]. When subjected to heat stress, NaHS-treated seedlings retained higher antioxidant enzyme activities and antioxidant levels (such as total glutathione and ascorbate) than controls. This suggested that pretreatment with NaHS could improve heat tolerance in maize seedlings [26]. Reduced glutathione concentrations may decrease under oxidative stress, when ROS formation increases, and thus, by boosting intracellular glutathione, H_2_S can significantly impact cell function, especially during periods of stress [135]. It showed that H_2_S in the form of NaHS improved maize seed germination under heat stress, as well as increased tissue viability and decreased malondialdehyde (MDA) accumulation caused by the application of heat [25]. NaHS acting as an H_2_S donor, subsequently improved salicyclic acid-induced heat tolerance in maize [145], an effect that was countered by the use of an H_2_S biosynthesis inhibitor or H_2_S scavenger. According to Li et al. [46], the H_2_S donor NaHS was applied to tobacco to improve cell culture viability during heat stress. Here, it was proposed that the effects were caused by extracellular calcium ions across plasma membrane, and that the effects inside cells were dependent on the activity of calmodulin, a common calcium-binding protein. In a different study, strawberry roots were subjected to an acute heat shock in both the presence and absence of NaHS. In H_2_S-treated tissues, MDA, H_2_O_2_, and NO were all reduced [146]. Ascorbate and glutathione metabolism, as well as the activation of the genes for catalase, superoxide dismutase, and the heat shock proteins (HSP70, HSP80, and HSP90), have all been implicated in the prevention of heat-induced tissue damage [146]. Wheat seedlings treated with foliar NaHS exhibited improved heat tolerance. Antioxidant levels increased, indicators of oxidative stress, such as MDA, decreased, and it was suggested that foliar application of H_2_S donors would be advantageous [45]. Under heat stress, hydrogen sulfide was found to increase glucose utilization and decrease heat-induced photosynthesis reduction in wheat [41]. Understanding how H_2_S impacts different tissues under different conditions, such as heat stress, is crucial. An overview of the various mechanisms involving H_2_S in regulating plant responses to heat stress as shown in Figure 2b.

### 2.4. Carbon Dioxide

The primary cause of global warming is the exponential increase in CO_2_ concentration in the atmosphere. The temperature has risen by 0.85 °C since pre-industrial times, along with a 129 ppm CO_2_ increase. The Intergovernmental Panel on Climate Change predicts that atmospheric CO_2_ levels will rise from their current level of 412 ppm to 936 ppm along with warmer weather, with temperature increases of up to 2.6 to 4.8 °C in extreme scenarios [3,147]. Regardless of any effects on the climate, rising CO_2_ concentrations have significant direct effects on plant growth, physiology, and chemistry [148]. The key function of CO_2_ in plant metabolism accounts for these effects. Agriculture producers worldwide use additional CO_2_ in their greenhouses to increase agricultural yields while enhancing the quality of their crops, which also experience heat stress [149]. When temperatures are high, plants frequently close their stomata, reducing CO_2_ flux and photosynthesis [150]. Additionally, under those circumstances, gas solubility rises, affecting the proximity of O_2_/CO_2_ to Rubisco active sites, affecting the availability of those gases and influencing photorespiration and respiration, changing the energetic metabolism of plants [151].

Numerous investigations with various plant species found that, in high-temperature environments, increased CO_2_ positively impacted photosynthesis and biomass yield [152,153,154,155,156]. In tall fescue, elevated CO_2_ increased both the rate of photosynthetic activity and resistance to heat stress [157]. According to a study conducted by Li X. et al. [48] elevated CO_2_ reduced heat stress in tomato plants by effectively regulating the cellular redox-balance in an ABA-independent manner. Through increased photosynthesis and water use efficiency, and reduced stomatal conductance and transpiration, elevated CO_2_ enhances plant growth and biomass [158,159]. Increasing CO_2_ reduces the impact of stressful conditions including heat and water stress [160,161]. According to Bauweraerts et al. [162], elevated CO_2_ lessens the detrimental effects of heat and water stresses on the photosynthetic parameters of *Quercus rubra* and *Pinus taeda*.

**Figure 3:**
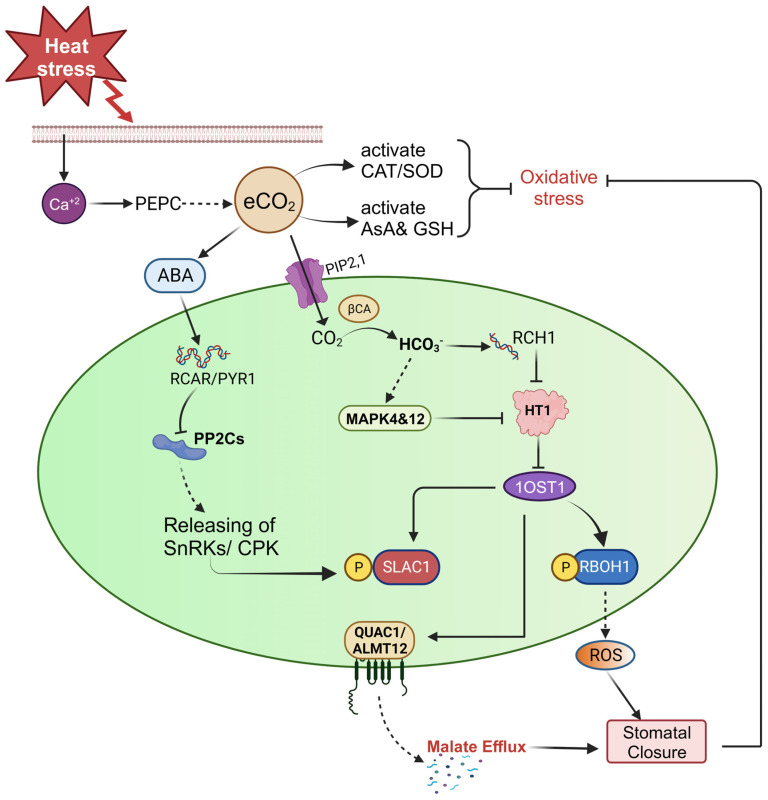
Under conditions of heat stress, the presence of calcium ions can trigger the activation of the PEPC (Phosphoenol pyruvate carboxylase) enzyme, which in turn increases the production of CO_2_. This elevated CO_2_ is then transported by the aquaporin PIP2;1 and converted into HCO_3_^−^ through the action of the β-CA (β-carbonic anhydrase) enzyme. One potential effect of CO_2_ is the activation of protein kinases 4&12 (MPK4&12) and RCH1 (Resistance to high CO_2_), which leads to the inhibition of HT1 (high leaf temperature 1). The suppression of HT1 activity subsequently deactivates 1OST1 (1open stomata 1). Furthermore, the elevated CO_2_ interacts with ABA, and ABA enters the guard cells to facilitate the closure of stomata by activating the RCAR/PYR1 (Regulatory components of ABA receptors/Pyrabactin resistance 1) receptors. This activation inhibits the PP2Cs protein phosphatases. In both cases of elevated CO_2_ and ABA, the SLAC1 (slow ion channel associated 1) ion channel is activated through the inhibition of OST1 and the action of SnRK2 (Ca^+2^ independent protein kinase) and CPK (calcium-dependent protein kinase). This activation results in the efflux of malate from ALMT12/QUAC1 (aluminum-activated malate transporter 12/quickly activating anion channel 1) and ultimately leads to the closure of stomata. Elevated CO_2_ also overcomes the effect of oxidative stress by stimulating the production of antioxidant enzymes and antioxidants.

Considering the likely future environmental conditions brought on by global climate change, researchers have been particularly interested in examining the interactions between high temperature, and elevated CO_2_ on the performance of crop plants (Figure 3). According to research, plants exposed to high CO_2_ concentrations can reduce the impact of heat stress [163,164]. The impact of CO_2_ on abiotic stress has been shown to vary significantly, and the underlying mechanisms are still undetermined. It is obvious that increased CO_2_ causes stomatal closure in addition to providing more carbon. Xu Z. et al. [165] stated that increased CO_2_ reduced stomatal conductance and controlled gene expression to counteract the negative effects of drought. Elevated CO_2_ helps plants tolerate high-temperature and scarce water situations by reducing oxidative stress and increasing water status of Arabidopsis. This reduction effect was constant across plant parameters [166]. The performance of plants, however, was not enhanced by elevated CO_2_ when temperatures were high. In comparison to plants under heat stress and ambient CO_2_, Yu et al. [163] found that *Festuca arundinacea* plants exposed to elevated CO_2_ and heat stress accumulated more metabolites like organic acids, amino acids, and carbohydrates. This led to improved growth, photosynthesis, and respiration. When compared to plants grown at ambient CO_2_, the combination of elevated CO_2_ and high temperature (from 25 °C to 42 °C) improved photochemical efficiency, energy use, and biochemical functioning in *Coffea arabica* and *Coffea canephora*, especially in the warmer condition [156,167]. According to Abebe et al. [168], increased grain yield, harvest index, cob length, crude protein content, and leaf area were some indicators of the beneficial effects of increased CO_2_ on temperature-stressed maize plants. Studies on coffee plants under heat stress have revealed increased antioxidant enzyme activity, which controls the excessive accumulation of ROS. Other genes, such as chaperonins and *HSP70*, were also up-regulated, helping to lessen heat stress and protect PS-II function [167]. Ramalho et al. [169] investigated the effect of heat and elevated CO_2_ on the quality of *Coffea arabica* beans. It was found that higher temperatures reduce bean quality, but this effect was mitigated by the interaction with increased CO_2_, which maintained bean properties nearer to or even better than those obtained under control conditions. Woody plants may benefit from increased by improving their photosynthetic apparatus, cell wall composition, and specialized metabolites that may be involved in stress signaling and defense [170]. Furthermore, Madan et al. [155] noticed that high CO_2_ exposure did not lessen the effects of heat stress on rice cultivars’ capacity to set seeds or produce grains.

### 2.5. Carbon Monoxide

Carbon monoxide (CO), which has one carbon and one oxygen atom, is tasteless, colorless, and odorless gas with low molecular weight of 28.01 g/mol. CO has emerged as a signaling molecule in plants due to its capacity to stimulate physiological processes such as seed germination, root development, and stomatal closure [171,172,173]. Wilks [174] was the first to discover CO biosynthesis in plants. Smaller plants at the soil’s surface and the soil-air interface are a significant source of light-independent CO gas [175]. CO is also produced by photosynthesis in living plants [176,177]. Additionally, it has been shown that heme methylene bonds can be disrupted and CO released when hydrogen peroxide (H_2_O_2_) or ascorbic acid is used [178]. Biochemical findings confirmed Heme oxygenase as a significant enzymatic source of endogenous CO production [179].

**Figure 4:**
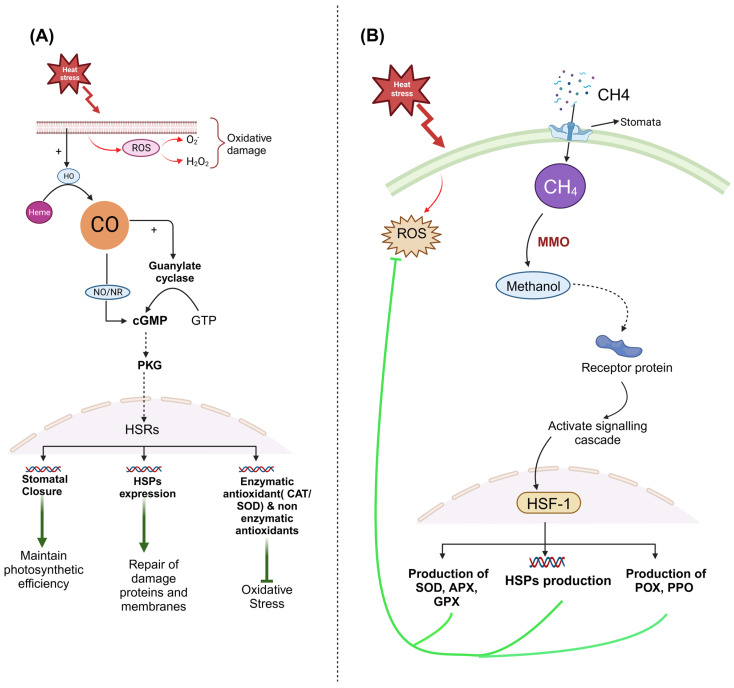
(**A**) Heat stress often leads to the formation of reactive oxygen species (ROS), such as superoxide radicals (O_2_-) and hydrogen peroxide (H_2_O_2_). In order to counteract the detrimental effects of ROS, plants activate heme oxygenase (HO), an enzyme responsible for converting heme to carbon monoxide (CO) as a byproduct. CO can then activate guanylate cyclase (GC), which triggers the production of cGMP. cGMP acts as a secondary messenger in various signaling pathways, including the stomatal signaling pathway. It activates the PKG (Protein Kinase-G) in guard cells, which are involved in phosphorylating different target proteins, including ion transporters and channels. Additionally, CO regulates the expression of heat shock proteins (HSPs), which play a crucial role in safeguarding proteins from denaturation and aggregation during stressful conditions. Furthermore, CO induces the production of both enzymatic (SOD/CAT) and non-enzymatic antioxidants to scavenge ROS production, thereby inhibiting oxidative stress. (**B**) A straightforward model is presented in this illustration to demonstrate the role of CH_4_ in a stressful environment. Methane penetrates the plant cell through stomata and is transformed into methanol by the enzymatic activity of MMO (Methane monooxygenase). Methanol is a toxic substance, but it also acts as a signaling molecule that can stimulate the production of HSPs. Methanol binds to a receptor protein on the surface of the (ER) and triggers a signaling cascade that results in the increased production of HSPs and the activation of antioxidant and oxidative enzymes. These enzymes are then transported to various parts of the cell, where they help to safeguard the cell from ROS damage.

CO is rapidly induced in plants by abiotic stresses and modulates plant responses to such stresses. CO has a significant impact on intracellular signaling mechanisms such as ensuring the maintenance of ROS equilibrium in the presence of oxidative stress. CO is crucial for intracellular redox signaling and the activation of antioxidant defense systems [180,181]. According to Cao et al. [172], CO is also required for the reduction of oxidative damage brought on by abiotic stress. Figure 4A illustrates a model of carbon monoxide-mediated signaling in plant responses to abiotic stress such as heat stress. Exogenous CO is toxic to plants and animals at high concentrations, but it plays an important role as a signaling mediator in many physiological processes at low concentrations [182]. The reduction of oxidative damage was caused by the induction of CAT and SOD activity by CO in aqueous solution [183]. There are currently limited studies on the role of CO in plant resilience to high-temperature stress. Hematin (CO donors) significantly improved the ability of cells to recover from heat stress and grow again, as well as reducing malondialdehyde accumulation and a decline in cell vitality. Hematin treatments also increased the activity of L-cysteine desulfhydrase, a crucial enzyme in the biosynthesis of H_2_S, which in turn caused tobacco cells to accumulate endogenous H_2_S. Therefore, it suggests that CO pretreatment could increase the heat tolerance of tobacco suspension-cultured cells [23]. In the case of other abiotic stresses, the administration of CO solution increased Indian mustard’s tolerance to mercury (Hg) and prevented the lipid peroxidation and root growth inhibition that was caused by Hg [184,185]. CO, together with other signaling molecules like as phytohormones, NO, and ROS, has a favorable effect on salt or heavy metal stresses [186]. Wheat seedling leaves may benefit from the exogenous application of low concentrations of CO donor hematin to protect them from salt-induced oxidative damage [187]. Wheat seedling roots experience a biphasic burst of CO production in response to NaCl, suggesting CO may be a crucial factor in the tolerance to salinity. CO may increase antioxidant system parameters and maintain ion homeostasis, both of which were partially mediated by NO signaling, thereby conferring increased tolerance to salinity stress in roots of wheat seedlings [188]. CO can increase a plant’s ability to withstand abiotic stress, but its precise biological functions signaling pathway in plants are largely unknown. Although there have been preliminary advances in our understanding of how CO regulates plant growth and development and the ability of plants to withstand environmental stresses, the field of CO research in heat stress tolerance is still in its infancy. Figure 4B demonstrates the role of CH_4_ in a stressful environment.

### 2.6. Methane

The second most prevalent greenhouse gas on the earth is methane (CH_4_). It has no colour, no smell, is safe, volatile, and is slightly soluble in water [19,189]. Methane is a tetrahedral molecule, which is present in both plants and animals, have been found to function as signaling molecule. CH_4_, a unique gaseous signal molecule, can regulate plant physiological processes such as seed germination, seedling growth, lateral rooting, adventitious root development, and post-harvest freshness [33,171,190,191,192]. CH_4_ has significant roles in the growth and environmental adaptation of plants. In addition, CH_4_ plays a crucial role as a key regulator in plants that are experiencing abiotic stress [190,193,194]. CH_4_ could improve plant abiotic stress resistance in general by strengthening the antioxidant defense system. According to several studies, plants can produce CH_4_ when the environment is aerobic [195]. The exact mechanism for CH_4_ production is not yet completely understood. It has been suggested that plants can produce CH_4_ through four different mechanisms. (1) By impeding the electron transport chain in the inner membrane of plant mitochondria, sodium azide causes the production of CH_4_. (2) Hydrogen peroxide oxidizes methionine to produce methionine sulfoxide. Methionine sulfoxide can demethylate its own to form methyl radicals by homogenizing the split-cleavage bond that releases CH_4_. (3) Under blue light, amino acids combine to form amino acid methyl. In canola, amino acid methyl is combined with ROS to produce CH_4_. (4) Under ultraviolet light, tryptophan produces singlet oxygen. Singlet oxygen can be converted to reactive hydroxyl radical in the presence of a biological reducing agent such as phenol. The hydroxyl radical and the methyl ester groups in pectin combine to form CH_4_ [196].

Higher plants under normal or stressful conditions could produce and release endogenous CH_4_ [193,197,198]. Various abiotic stresses, such as ultraviolet radiation [199,200], high temperatures [201], and heavy metal stress [197], are thought to cause plants to produce more CH_4_. According to a recent study, ROS could increase the production of endogenous CH_4_ [198]. It has been demonstrated that CH_4_ protects against ROS, functions as a signaling molecule, and controls numerous genes in plants to influence their growth and development [196]. According to biochemical and molecular research, CH_4_ can reduce the toxicity of heavy metals like cadmium and copper to plants [194], improve the salt tolerance of alfalfa (*Medicago sativa*) [190], and support maize’s capacity to resist osmotic stress [193]. The protective effects of CH_4_ on different abiotic stresses in plants have been confirmed by research. CH_4_ reduced the toxicity of heavy metals and osmotic stress primarily by boosting the activity of key antioxidant enzymes and by restoring redox equilibrium [33,202]. Through partially raising heme oxygenase-1 (HO-1) expression, boosting the antioxidant response, and changing K^+^/Na^+^ ion balance, CH_4_ may help plants tolerate salt stress [190]. Overall, it has been discovered that exposure to CH_4_ is associated with an increase in the gene expression and activity of antioxidant enzymes, which restores redox equilibrium. However, the mechanisms by which CH_4_ serves when exposed to high temperatures are not yet understood. high-temperature stress, further studies must be conducted further studies must be conducted in order to better understand the mechanisms that support CH4 under high-temperature stress. Figure 4B presents a simple model to illustrate the function of CH_4_ in a heat-stressed condition.

## 3. Crosstalk of Gaseous Molecules

According to findings from earlier studies, crosstalk between NO and H_2_S exists in the acquisition of abiotic tolerance, like heat stress. Exogenous NO pretreatment improved maize seedling survival rates under heat stress, and NO raised H_2_S content by boosting L-DES activity. H_2_S may act downstream of the NO signal in NO-induced heat tolerance, as shown by the fact that H_2_S synthesis inhibitors and an H_2_S scavenger completely reversed NO-induced heat tolerance [203]. Similar to this, Li Z.G. et al. [204] found that H_2_O_2_ pretreatment increased L-DES activity, which in turn increased the amount of endogenous H_2_S and improved maize seedling heat tolerance. This effect was further enhanced by the addition of SNP and NaHS. The ROS-scavenging system may have played a significant part in the NO and H_2_S crosstalk-evoked thermo-tolerance in maize seedlings as NO and H_2_S crosstalk increased the activity of the ROS-scavenging system in plants [205]. Additionally, ethylene applied as a foliar spray improved rice seedlings thermo-tolerance by modulating the activity of antioxidant enzymes, osmolytes, and photosynthetic metabolism through cross-talk with NO and H_2_S [39]. Wheat seedling thermo-tolerance is improved by NO and H_2_S crosstalk which reduces glucose sensitivity and oxidative stress via the AsA-GSH cycle [41]. Chinese cabbage and Poplar (*Populus trichocarpa*) plants exhibit crosstalk between NO and H_2_S in the development of heat tolerance caused by H_2_S and NO [206,207]. Despite numerous lines of evidence linking NO to heat tolerance, the interaction between NO and ethylene under heat stress has not been thoroughly studied [10]. However, *Medicago sativa* plantlets exposed for two hours at 37 °C produced more NO and released less ethylene in alfalfa [208]. There is a possibility of a functional interaction between ethylene, H_2_S, and S in relation to the ability to withstand heat stress because the biosynthesis of ethylene and H_2_S is linked to the S-assimilation pathway. In Brassica, ethylene was shown to boost ATP-S activity and S absorption, and it was also discovered to cause H_2_S production in Arabidopsis leaves by increasing L-/D-cysteine desulfhydrase activity [209,210]. Findings of Pan et al. [68] provide strong evidence that increased CO_2_-induced heat stress response in tomato plants depends on ethylene production and signaling. Furthermore, elevated CO_2_-induced ethylene and *ERF1* may promote *HSFA2* to activate the transcription of HSPs genes, increasing tomato plants’ resistance to heat stress [68]. The induction of several ethylene signaling and synthesis genes by increased CO_2_ was consistent with earlier research [211].

CO shows crosstalk with other gas signaling molecules like NO, H_2_S, H_2_, and CH_4_ as well as phytohormones like IAA, ABA, and GA [12]. The ability of wheat seedling roots to produce NO after daily exogenous CO treatment suggests that NO may be a component of the CO action’s downstream signal molecule [11]. There are not numerous studies on interactions between CO and H_2_S. The formation of adventitious roots may be specifically influenced by H_2_S, which may also encourage the generation of CO, which in turn increases the growth of lateral roots [212]. CO increases cellular heat resistance in tobacco plants, but NaHS increases CO-induced heat resistance. However, both effects can be reduced by PAG, a particular inhibitor of H_2_S biosynthesis, or by HT, a scavenger of H_2_S [23]. CH_4_-induced Cd tolerance in alfalfa seedlings requires DES-dependent H_2_S signaling via the reduction of cadmium ion inflow and accumulation and the formation of glutathione homeostasis and antioxidant defense [213]. In cucumber, both NO and CO signaling pathways were engaged in CH_4_-induced adventitious root development [171,214]. NO could play a function in high CO_2_-induced flavonoid production by connecting the SA pathway [215]. Evidence suggests that NO-dependent abiotic stress tolerance generated by CH_4_ may involve NR and NOS-like proteins [202].

## 4. Conclusions and Future Perspectives

The majority of study on a variety of gaseous molecules has been focused on ethylene, nitric oxide (NO), hydrogen sulfide (H_2_S), and to a lesser extent on carbon monoxide (CO), methane (CH_4_) and carbon dioxide (CO_2_). These molecules can quickly diffuse and modify cellular compartments due to their gaseous nature. Gaseous molecules can interact with one another, plant hormones, nutrients, ions, and polyamines, therefore efficiently reducing plant stress by influencing various defense mechanisms in plants. The main function of gaseous molecules largely depends on their concentration, signaling, and crosstalk with other molecules. The reports that are currently available indicate that these gaseous molecules are released in plants under various adverse circumstances. Importantly, these gaseous molecules increase the ability of plants to withstand a range of environmental stimuli. They do this primarily by controlling the activity of antioxidant enzymes, reducing oxidative stress and lipid peroxidation, maintaining ion homeostasis, and restoring glutathione homeostasis. Future research on the biosynthesis of these gaseous molecules should concentrate on the molecular specifics of their production routes in plants under abiotic stress, such as high temperature stress. In animals, carbon monoxide has been extensively studied as a gaseous signaling molecule, but research on CO in plants is still in its initial stages. In connection to the interaction with other signaling molecules, CO can increase plant abiotic stress tolerance, although its precise biological functions in plants and its precise signaling pathway remain largely unclear. Exogenous application of relatively low concentrations of H_2_S donors via spraying or fumigation consistently shows the positive impact of H_2_S on plant growth performance under different environmental stresses. H_2_S may have a significant additional value for the use and advancement in modern agriculture, particularly considering the low cost of these compounds and the ease of their application. Focusing on discovering ethylene’s opposing and beneficial interactions with other signaling indications in the future will help us learn more about how ethylene interacts with various gaseous molecules and environmental factors. Significant work has been done in understanding the mechanism and signaling pathways that govern the achievement of thermo-tolerance in plants. It is still necessary to clarify the role of ethylene and other gaseous molecules in regulating biochemical and molecular processes for plant protection against heat stress. Additionally, the information that is currently accessible may help to strengthen the mechanism for minimizing heat stress damage, particularly in plants with agronomic significance.

## Figures and Tables

**Table 1:** Reports on various plant species under high-temperature stress and the effects of applying exogenous gaseous molecules.

Plant Species	Source of Signaling Molecule	Tissue Exposed	High-Temperature Stress	Observed Effects	References
Rice (*Oryza sativa* L.)	Ethylene; 10 μM ACC for 1 h	12 days seedlings	45 °C for 4 days	Enhanced HSFs (*HSFA1a*, *HSFA2a, c, d, e,* and *f*) and ethylene gene expression *ACC oxidase 1* and *ACC oxidase 3*, *EIN 2*, *EIN-like 1*, and *EIN-like 2.*	[37]
Wheat (*Triticum aestivum* L.)	Ethylene; 200 µL L^−1^ ethephon	Seedlings	40 °C for 15 days	Enhanced proline accumulation and activity of antioxidant enzymes, SOD, APX and GR.	[38]
Rice (*Oryza sativa* L.)	Ethylene; 1.6 mM ethephon	15 days seedlings	40 °C for 15 days	Increased photosynthesis by up-regulating the photosystem II *psbA* and *psbB* genes.	[39]
*Arabidopsis thaliana*	Ethylene; 10 µM ACC	10 days seedlings	42 °C for 4 h	With the the ethylene signaling pathway, myo inositol phosphate synthase regulates photosynthetic efficiency and chlorophyll content.	[40]
Wheat (*Triticum aestivum* L.)	NO; 100 μM SNP	Seedlings	40 °C for 6 h	Increased net photosynthesis, chlorophyll content, intercellular CO_2_ concentration, Rubisco activity, and stomatal conductance.	[41]
Strawberry (*Fragaria* × *ananassa* Duch.)	NO; 0, 50, 100 μM SNP	Seedlings	40 °C for 2, 5 and 10 h	Increased the expression of *FaTHSFA2a* and *FaTHSFB1a*, as well as the expression of *HSP70* and *HSP90.*	[42]
Mung bean (*Phaseolus radiatus*)	NO; 150 μM SNP	Leaf discs	45 °C for 90 min	Reduced lipid peroxidation, increased Fv/Fm, increased MnSOD, CuSOD, and FeSOD activity.	[43]
Lentil (*Lens culinaris* Medik.)	NO; 1 mM SNP	Seedlings	32 °C for 12 h	Pollen grain germination and viability, stigma receptivity, and ovular viability increased significantly.	[44]
Wheat (*Triticum aestivum* L.)	H_2_S; 0–1.5 mmol L^–1^ NaHS	Seedlings	38 °C for 24 h	Increased soluble sugar content, SOD, and APX activities.	[45]
Maize (*Zea mays* L.)	H_2_S; 0.5 mM NaHS	Seeds	48 °C for 18 h	Significantly increased survival percentage of seeds and survival percentage of seedlings.	[25]
Tobacco(*Nicotiana tabacum* L.)	H_2_S; 50 μM NaHS	Calli (young stem)	43 °C for 1, 3, 5 or 7 h	Increased survival percentage of tobacco suspension cells and reduced MDA accumulation.	[46]
Maize (*Zea mays* L.)	H_2_S; 0.5 mM NaHS	Seeds	47 °C for 15 h	Improved the activity of BADH and endogenous betaine accumulation,	[47]
Tomato (*Solanum lycopersicum* L.)	800 μmol·mol^−1^ CO_2_	Seedlings	42 °C for 24 h	Lowered electrolyte leakage and MDA levels, increased Fv/Fm value, and increased antioxidant enzyme activity	[48]
Wheat (*Triticum aestivum* L.)	780 mmol L^−1^ CO_2_	Seedlings	42 °C for 3 days	Increased concentration of sucrose, glucose, and fructose and increased photosynthetic rate and grain yield	[49]
Cowpea (*Vigna unguicuiata* L.)	700 μmol mol^−1^ CO_2_	Whole plant	30 °C	Increased carbohydrate content (starch in leaves, stems, and peduncles)	[50]
Bermudagrass (*Cynodon dactylon* Pers.)	800 μmol·mol^−1^ CO_2_	Stolons	45 °C	Increased *Pn*, Chl and Fv/Fm. Improved metabolic pathways involved in the, fructose light reaction (ATP synthase subunit and PS I reaction center subunit), carbon fixation of photosynthesis (GAPDH, FBA, PGK, SBPase, and sugars), and glycolysis (GAPDH, glucose, fructose, and galactose).	[51]
Wheat (*Triticum aestivum* L.)	CO; 5 µM hemin	Seedlings	45 °C for 10 min	NO levels in root seedlings have increased. Hemin-induced antioxidant enzyme activation (superoxide dismutase, catalase, and guaiacol peroxidase)	[52]

## Data Availability

Not applicable.
